# Supplementary material for: Complexome profiling of the Chlamydomonas psb28 mutant reveals TEF5 as an early PSII assembly factor
Source: Plant Cell. 2025 Mar 17;37(6):koaf055. doi: 10.1093/plcell/koaf055 (PMC12164590; doi:10.1093/plcell/koaf055)
Supplement: koaf055_Supplementary_Data [file koaf055_supplementary_data.zip › psb28_interactive.zip!/plots/intensity/qualityPlots/peptides/LHCBM6_Cre06.g285250.t1.2.html]

### Cre06.g285250.t1.2

Trivial name: LHCBM6  
SeqCoverage [median]: 63.20  
Mass: 26.96 kDa  
  
 

| Sequence | MissedCleavage | Mass [Da] | wtIntensity | muIntensity | Score |
| --- | --- | --- | --- | --- | --- |
| GPVQNLDDHLANPTVNNAFAFATK | 0 | 2553.26 | 1950025.86 | 1286821.70 | 265.05 |
| GPVQNLDDHLANPTVNNAFAFATKFTPSA | 1 | 3056.50 | 1101.99 | 0.00 | 41.13 |
|  |  |  | 1951127.8 | 1286821.7 |  |
